# Deep Learning Methods Using Imagery from a Smartphone for Recognizing Sorghum Panicles and Counting Grains at a Plant Level

**DOI:** 10.34133/plantphenomics.0234

**Published:** 2024-08-28

**Authors:** Gustavo N. Santiago, Pedro H. Cisdeli Magalhaes, Ana J. P. Carcedo, Lucia Marziotte, Laura Mayor, Ignacio A. Ciampitti

**Affiliations:** ^1^ Department of Agronomy, Kansas State University, Manhattan, KS 66506, USA.; ^2^ Corteva Agriscience, Wamego, KS 66547, USA.; ^3^ Institute of Digital Agriculture and Advanced Analytics, Kansas State University, Manhattan, KS 66506, USA.

## Abstract

High-throughput phenotyping is the bottleneck for advancing field trait characterization and yield improvement in major field crops. Specifically for sorghum (*Sorghum bicolor* L.), rapid plant-level yield estimation is highly dependent on characterizing the number of grains within a panicle. In this context, the integration of computer vision and artificial intelligence algorithms with traditional field phenotyping can be a critical solution to reduce labor costs and time. Therefore, this study aims to improve sorghum panicle detection and grain number estimation from smartphone-capture images under field conditions. A preharvest benchmark dataset was collected at field scale (2023 season, Kansas, USA), with 648 images of sorghum panicles retrieved via smartphone device, and grain number counted. Each sorghum panicle image was manually labeled, and the images were augmented. Two models were trained using the Detectron2 and Yolov8 frameworks for detection and segmentation, with an average precision of 75% and 89%, respectively. For the grain number, 3 models were trained: MCNN (multiscale convolutional neural network), TCNN-Seed (two-column CNN-Seed), and Sorghum-Net (developed in this study). The Sorghum-Net model showed a mean absolute percentage error of 17%, surpassing the other models. Lastly, a simple equation was presented to relate the count from the model (using images from only one side of the panicle) to the field-derived observed number of grains per sorghum panicle. The resulting framework obtained an estimation of grain number with a 17% error. The proposed framework lays the foundation for the development of a more robust application to estimate sorghum yield using images from a smartphone at the plant level.

## Introduction

Sorghum (*Sorghum bicolor* L.) plays a pivotal role in global food security, serving as a vital source of nutrition for both humans and livestock in arid regions [1,2]. Accurate production estimates are crucial to informed decision making by farmers, guiding choices on crop and financial management decisions [3]. In addition, yield predictions are essential for breeders when selecting new genotypes. However, the conventional method of sorghum yield estimation relies on labor-intensive and time-consuming manual sampling at the plant level, which introduces the potential for human error, especially in large areas or with a large number of plots [4].

To address these challenges, there is a pressing need to develop high-throughput phenotyping methods to automate sorghum yield estimation. Grain number is a key component of sorghum yield [5], with a few attempts to estimate grain numbers using traditional and image-based approaches but have not been effectively translated to field scale [6–8]. A promising alternative is to use computer vision algorithms to extract meaningful information from images and videos [9,10]. Computer vision encompasses computational techniques for retrieving desired data through image processing [11], with convolutional neural networks (CNNs) being a notable image processing method inspired by the human neural system [12].

More recently, the application of CNN techniques in agriculture, especially for high-throughput phenotyping, has gained traction. Arya et al. [13] explored how deep learning and CNN techniques can help in plant phenotyping to speed up the current plant trait identification and selection process. Li et al., James et al., and Patel et al. [14–16] developed 3D, x-ray computed tomography, point cloud, and CNN models that are relevant for analyzing phenotypic aspects of sorghum plants, such as panicle architecture, grain number, and organ segmentation. However, a major challenge for many of these methods is the effective and pragmatic translation of these efforts to field conditions. To bring these techniques to the field scale, Young et al. [17] developed a ground robot capable of performing high-throughput phenotyping of sorghum plants. However, the relatively high cost of this technology remains a limitation to scalability and adoption.

With the aim of estimating sorghum yield production, several studies have utilized CNN models, such as 2-stage CNN [18], U-Net [19], weakly supervised frameworks [20], RetinaNet [21], and ResNet-50 [22], to accurately count sorghum panicles from imagery, achieving impressive results, often exceeding 95% accuracy. However, these studies predominantly relied on imagery obtained through unmanned aerial vehicles (UAVs), which presents challenges such as high equipment costs and the need for specialized training. To overcome these limitations, the use of smartphones, with their increasing computing power, embedded sensors, and portability [23], offers a viable solution. The current trend of using smartphone cameras in agriculture has seen success in various applications, including estimating plant nitrogen content and plant health in different crops [24–26]. However, efforts to estimate sorghum yield using smartphones under field conditions have been limited, with previous attempts using traditional image manipulation techniques [6,8].

This study aims to address this research knowledge gap by using CNNs to effectively remove background under plant-level conditions and count sorghum grains. Therefore, the specific objectives include (a) detecting and segmenting sorghum panicles at the field level using images from a smartphone camera, (b) using segmented images to count the number of grains within manually segmented panicles, and (c) using a linear model to estimate the total number of grains within a panicle based on the previous count. Through this innovative approach, we aim to provide a practical and cost-effective solution to pave the way for the development of a rapid sorghum yield estimation at the plant level, advancing agronomy and computer science.

## Materials and Methods

**Fig. 1.**
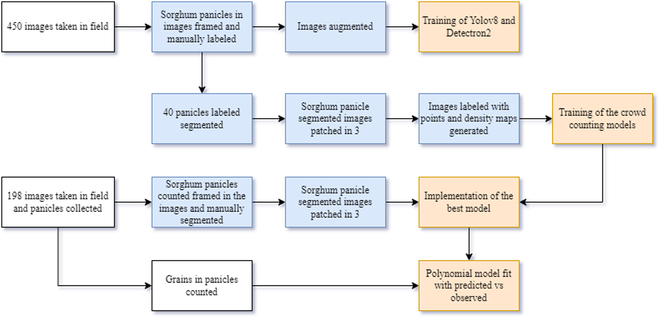
Research diagram of the proposed framework. The white boxes denote the manual component under field conditions, the blue boxes represent the processes executed via the computer, and the yellow boxes refer to the processes linked to machine learning models.

The study has been conducted employing a Lenovo Legion desktop computer with an Intel Core i7-10700 CPU @ 2.90GHz, NVIDIA GeForce RTX 2070 SUPER 8GB; 16GB RAM using CUDA 11.8 and Python 3.9. The overall description of the proposed framework is presented in Fig. 1.

### Detection and segmentation

#### Data

**Fig. 2.**
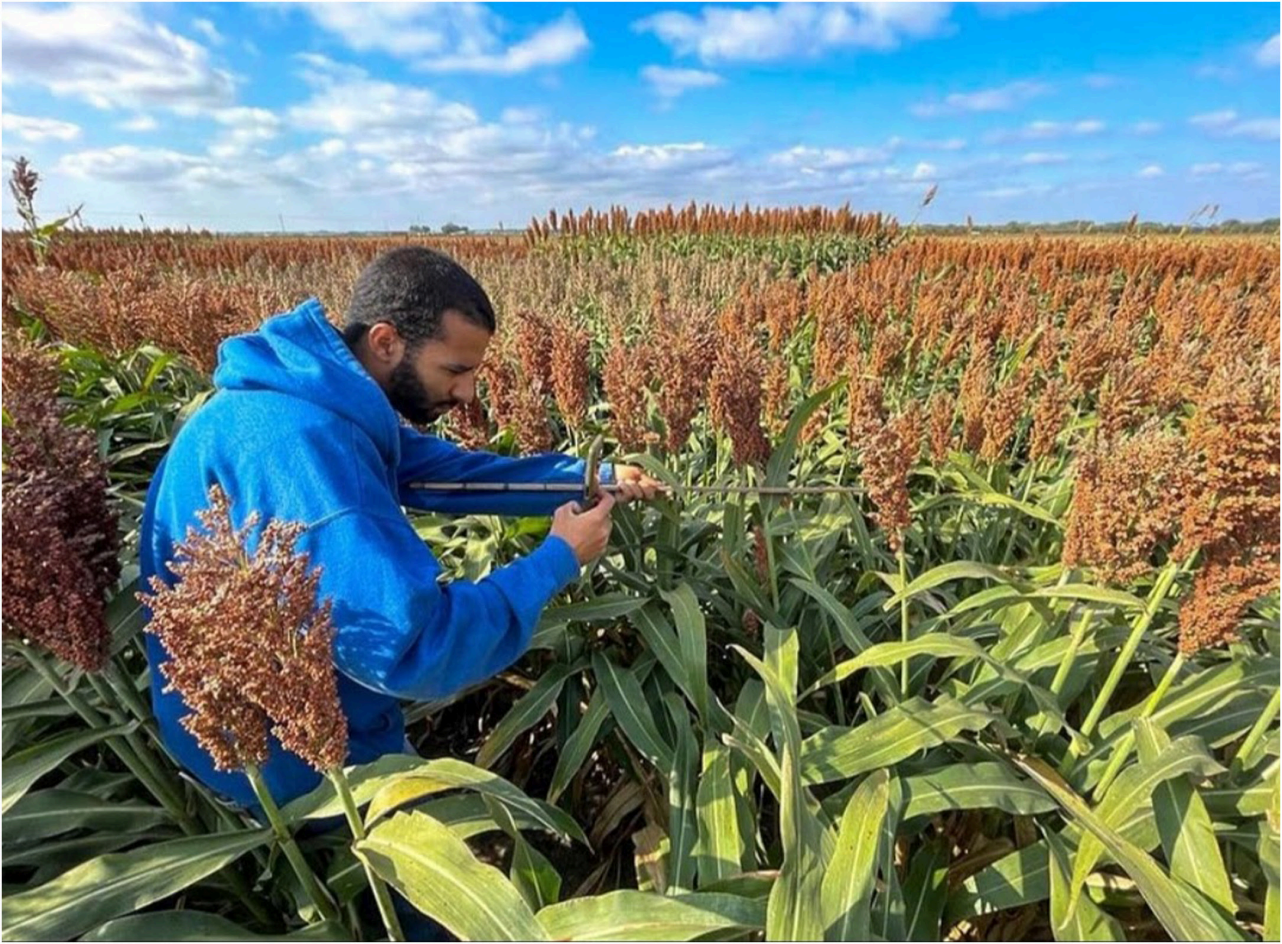
Photograph showing how the pictures were collected under field conditions.

A total of 457 images of sorghum panicles from 20 commercial hybrids were obtained during the preharvest (2023 October 14; 128 d after sowing), at an experimental plot located at Wamego, Kansas, USA from 9 AM to 12 AM. The 20 commercial sorghum hybrids encompassed a wide variability in their canopy and panicle architecture, panicle colors, and maturity groups, including 18 genotypes proceeding from the US breeding program and 2 tropical hybrids from the Mexican breeding program. The images centered on a single panicle at a distance of 1 m and parallel to the panicle, with other sorghum plants in the background. A generic smartphone was used with a camera of 64MP primary camera based on Sony IMX 582 Quad-Bayer 1/2” sensor with 0.8-μm pixels, 25-mm f/1.8 lens, set to automatic mode of image capture and saved in JPEG file extension. In all images, the sunlight was in the same direction as the camera to avoid high brightness in the camera sensor, and the distance to the panicles was 1 m, as shown in Fig. 2.

**Fig. 3.**
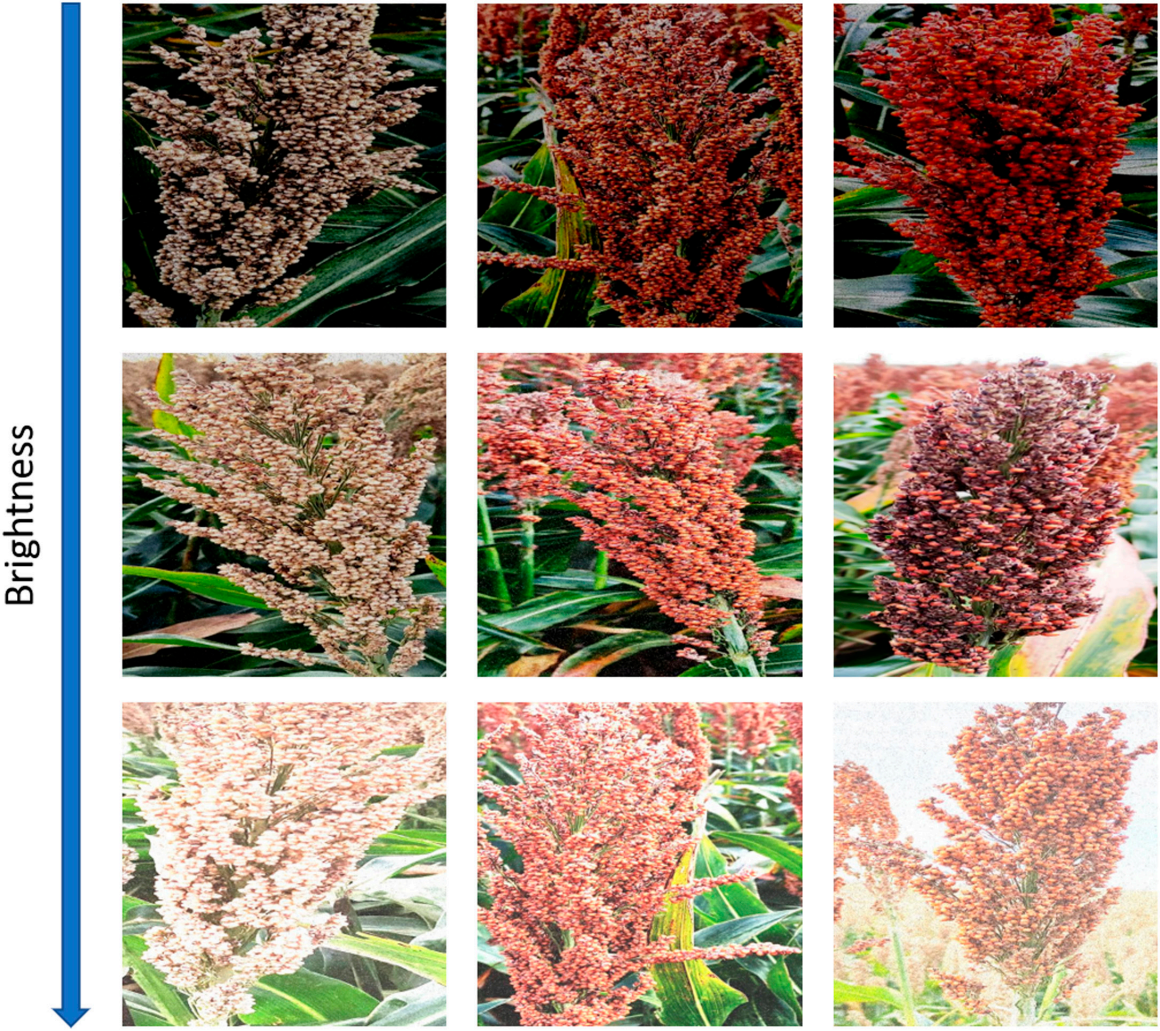
Examples of the dataset used in this study: sorghum panicles with different architecture, color, maturity, and panicle density. From top to bottom are images with crescent brightness. In the first column, there are white panicles; in the second one, there are red panicles; and in the third one, there are panicles with different densities. All the images show a different panicle architecture.

Using Roboflow software [27], all panicles in the framed images were manually labeled with polygons. The only class considered in this study was “panicles”, which refers only to the sorghum panicles, excluding the rest. An augmentation step was used to increase the image dataset. The images were subjected to random horizontal and vertical flipping, horizontal and vertical shifting, color and brightness modifications, zooming, and random cropping. Finally, the complete dataset resulted in 4,623 images of 640 × 640 pixels in size, with 4,500 for training, 102 for validation, and 21 for visual inspection. Some examples of the images from the resulting dataset are shown in Fig. 3.

#### Frameworks, parameters, and metrics

Two frameworks were compared in this step: Detectron2 [28] and Yolov8 [29]. Detectron2 was developed by Meta AI Research as the successor of Detectron and maskrcnn-benchmark and has several pretrained models for different computer vision applications. The Detectron2 framework includes models such as Faster R-CNN [9], Mask R-CNN [30], RetinaNet [21], DensePose [31], and Cascade R-CNN [32]. The Detectron2 CNN architecture used in this study was Mask R-CNN [30]. Yolov8, a member of the You Only Look Once (YOLO) family, was developed by Ultralytics for detection, segmentation, classification, pose, and tracking tasks. It has models pretrained on COCO [33], Open Image V7, and ImageNet [34] datasets. Further details on all model architectures can be found in the references presented in this section.

The following parameters were employed to train the models for the detection and segmentation in both frameworks: 28 epochs, a learning rate of 0.001, and a batch size of 8. For the other parameters, default values from the frameworks were employed. The pretrained weights mask_rcnn_R_101_FPN_3x for Detectron2 and yolov8x-seg for Yolov8 were used to support the training process. The model outputs from the training were evaluated using the following metrics: average precision with 50% of the region compared to the ground truth (AP50) and average recall (AR), which were obtained directly from the frameworks. Finally, a visual inspection of 21 images from the dataset was performed to evaluate the overall performance of the segmentation task.

### Density maps

#### Data

From the 457 images collected in the field, 40 images were randomly selected. The framed panicle within each image was manually segmented, excluding the background. Manual segmentation was used to ensure that the panicle of interest was perfectly segmented. The images were then cropped into 3 equal height areas: the top, middle, and bottom of the panicle. This step was necessary due to the large number of grains within a panicle (up to 4,000 grains) and their small size (~8 pixels). This procedure resulted in 120 images.

Following the methodology proposed by Zhang et al. [35], density maps were used to obtain the number of grains because generally, detection methods have poor performance in detecting small objects [36] and poor stability and scalability [37]. Foreground segmentation is challenging, and an inaccurate segmentation could undermine the final count. The density maps provide the distribution of the number of grains in the image, allowing the researcher to corroborate the output performance.

The ground truth density maps were created by labeling the images by placing a point in each visible grain using the LabelMe software. This process generated a JSON file containing the point coordinates for each picture. The images and the point coordinates were then resized to 224 × 224. The density maps were created using the point coordinates and the function gaussian_filter from scipy [38] with a sigma value of 5. The function sum from the numpy library [39] was employed to calculate the integral of all the pixels in the density map image and obtain the total amount of grains within an image. The images and the ground truth were augmented using imgaug [40] and opencv [41] libraries by changing the hue and saturation, adding Gaussian noise and blur, inverting color channels, changing contrast, adding value to each pixel, rotating, and flipping the images. At the end of the augmentation process, the dataset was composed of 4,800 images, which were split into 80% training and 20% testing.

#### Model architectures, parameters, and metrics

To train the models, 3 different architectures were employed: MCNN (multiscale convolutional neural network) [35], TCNN-Seed (two-column CNN-Seed) [37], and Sorghum-Net. The MCNN is a multicolumn CNN developed to count the number of people in an image, obtaining a good performance in crowd images. The TCNN-Seed was developed to count the number of seeds based on pod images. TCNN-Seed is a 2-column CNN that presents low error and a lower number of parameters, compared to MCNN. For more information regarding the architecture of these 2 CNN models, please refer to the original literature. Sorghum-Net is a multicolumn CNN that was specifically developed for this study, composed of 3 columns with 4 convolutional layers each, a concatenation, an up-sampling, and a convolutional layer at the end. The first column has a kernel size of 3 × 3 with convolutional layer dimensions of 80, 160, 80, and 40. The second column has a kernel size of 5 × 5 with convolutional layer dimensions of 40, 80, 40, and 20. The third column has a kernel size of 7 × 7 with convolutional layer dimensions of 20, 40, 20, and 40. This model architecture uses batch normalization after each convolutional layer of the columns and in front of the activation function. The activation function is the rectified linear unit. A max-pooling layer of a 2 × 2 size kernel with strides of 2 was used after the 2 first convolutional layers in each column.

**Fig. 4.**
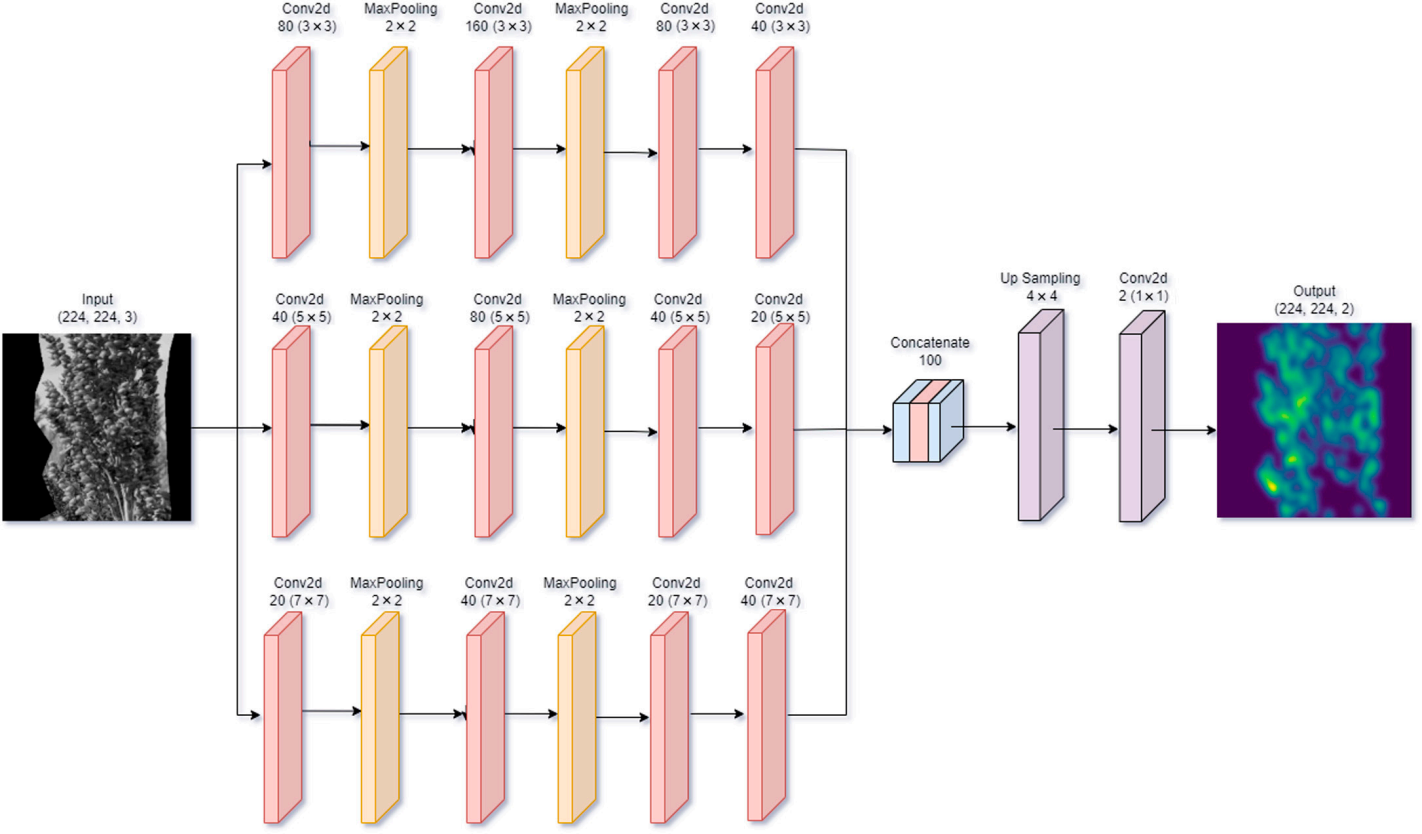
Overview of the Sorghum-Net architecture. The diagram represents the process from the segmented panicle to the density map as the final output, red boxes represent Conv2d, and yellow boxes represent MaxPooling. The TensorFlow framework was used to create and train the models. The parameters to train the models were set to a maximum epoch number of 200 with a patience of 20 epochs, batch size of 8, a learning rate of 0.001, the optimizer employed was ‘Adam’, and a loss function of MSE.

To summarize, the columns presented similar structures as follows: Conv2d-MaxPooling-Conv2d-MaxPooling-Conv2d-Conv2d-Conv2d. After the parallel columns, the feature map was concatenated and up-sampled to have the same size as the ground-truth density maps (224 × 224), and the up-sampled was used as input to a convolutional layer of 1 × 1 kernel size and a dimension of 2. The Sorghum-Net architecture is illustrated in Fig. 4.

The metrics used to compare the different models were: mean absolute error (MAE) (Eq. 1), mean squared error (MSE) (Eq. 2), and mean absolute percentage error (MAPE) (Eq. 3), from the scipy library [42]. The values used in the metrics were obtained by comparing the number of grains predicted by integrating the pixels of the output density maps from the models and the number of grains observed (manually counted) in the respective imagery from the test dataset.

$$\text{MAE}=\frac{1}{n}\sum_{i=1}^{n}|y_{i}-{\hat{y}}_{i}|$$ (1)

$$\text{MSE}=\frac{1}{n}\sum_{i=1}^{n}{\left(y_{i}-{\hat{y}}_{i}\right)}^{2}$$ (2)

$$\text{MAPE}=\left(\frac{1}{n}\sum_{i=1}^{n}\frac{|y_{i}-{\hat{y}}_{i}|}{y_{i}}\right)*100$$ (3)

where *n* is the number of observations, *y_i_* is the actual value of the *i*^th^ observation, and ${\hat{y}}_{i}$ is the predicted value of the *i*^th^ observation.

### Estimation of the panicle grain number

A different dataset of 198 images of sorghum panicles from those sorghum hybrids was obtained on the same day and using the same equipment, as explained in “Data” in the “Detection and segmentation” section. In this case, 2 images of the same panicle were taken from opposite sides. The panicles were then harvested and dried at 65°C until stable weight. The grains were removed from the panicles and counted using a machine to count grains.

Since a single image cannot represent the total grain number of a panicle, a model was fitted to estimate the total number of grains from the density map estimations. Following the procedures of “Data” in the “Density maps” section, the panicles were manually segmented, the background was removed, and the imagery was patched in 3. The output imagery was then input into the best model from the “Density maps” section (Sorghum-Net), and the number of grains in the image was estimated by the integral of the pixels in the image using the sum function from the numpy library [39]. The total grains of a single image were set as the sum of the grains of the 3 patches.

To compare the symmetry of the panicles, the 2 images of the same panicle on opposite sides were compared using the metrics MAE, MSE, and root mean square error (RMSE) (Eq. 4), employing the scikit-learn library [43]. A polynomial linear regression model was fitted using the scikit-learn library [43], with the observed counted grains as dependent and the estimation from the density maps as independent, split in a random 70:30% train: test proportion. Finally, to quantify the accuracy of the model, the scikit-learn library [43] was employed to calculate the following metrics: coefficient of determination (*R*^2^), MSE, RMSE, and MAPE.

$$\text{RMSE}=\sqrt{\frac{1}{n}\sum_{i=1}^{n}{\left(y_{i}-{\hat{y}}_{i}\right)}^{2}}$$ (4)

## Results

### Detection and segmentation

The model trained using the Yolov8 framework performed better in all evaluated parameters (Table 1). Yolov8 performed better in correctly classifying a sample as positive (Table 1; AP50, 0.887 and 0.890 versus 0.785 and 0.756), faster (Table 1; inference time, 0.719 s versus 0.925 s), and with a smaller model file size (Table 1; model weight, 141MB versus 491MB).

**Table 1.** Detection and segmentation models’ comparison. Metrics were obtained employing the frameworks Detectron2 and Yolov8 to detect and segment panicles in imagery obtained at field scale using a smartphone camera.

Framework	Detection		Segmentation		Inference time (s)	Weights file size (MB)
	AP50	AR	AP50	AR		
Detectron2 (Meta Research[28])	0.785	0.733	0.756	0.652	0.925	491
Yolov8 (Jocher et al. [29])	0.887	0.789	0.890	0.793	0.719	141

**Fig. 5.**
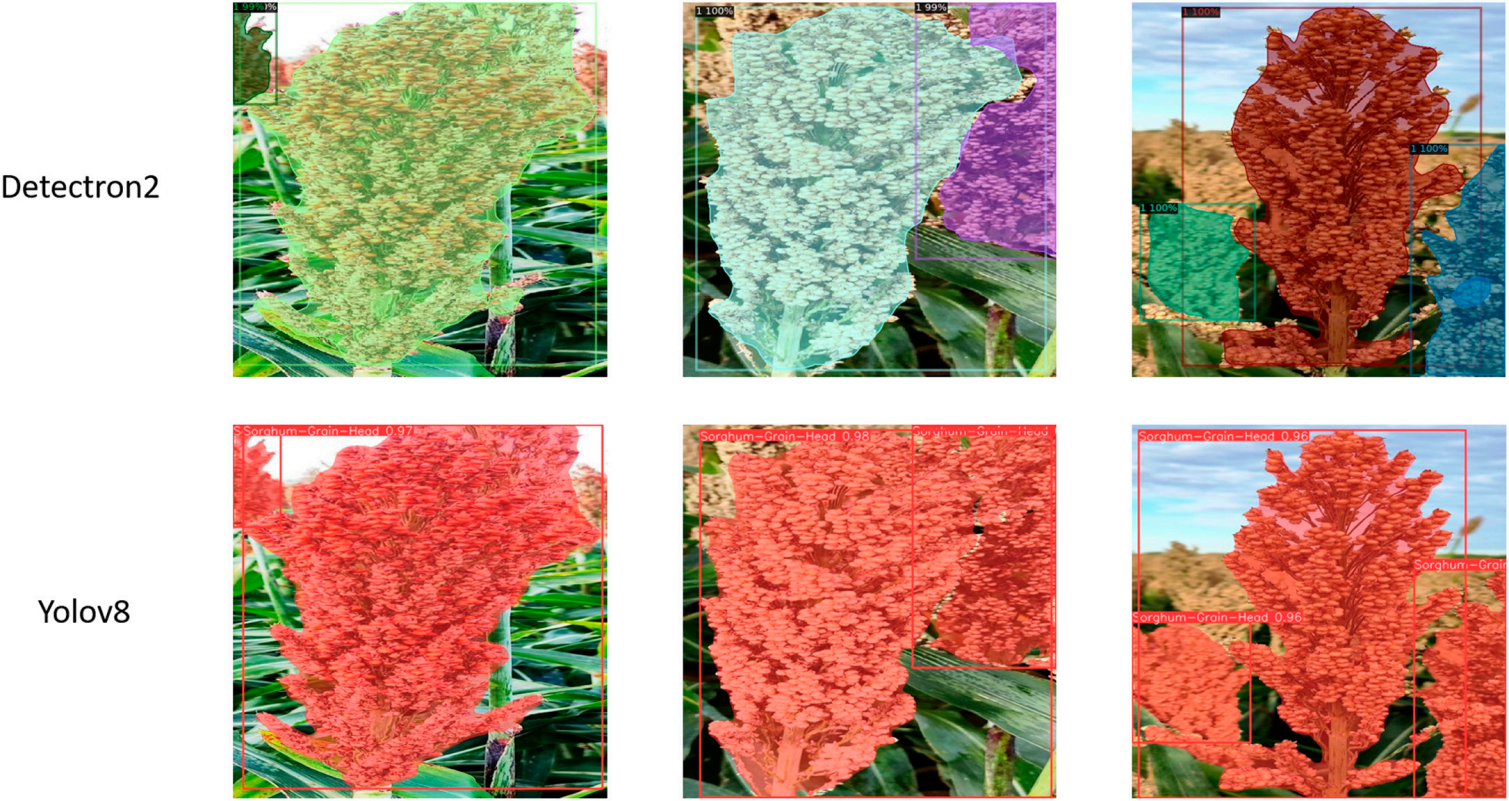
Sorghum panicle segmentation. Predictions were performed by the models trained using the frameworks Detectron2 and Yolov8 over sorghum images.

Three examples were chosen to illustrate different scenarios for field conditions (Fig. 5). Figure 5A shows a partially true positive, with both models (Detectron2 and Yolo8) detecting and segmenting the panicle of interest but also considering part of the background for the images. Figure 5B shows a partially adequate identification of the panicle for both models; however, the model using Yolov8 presented higher accuracy, considering more parts of the same panicle. Figure 5C shows a situation where was possible to see 3 panicles in the imagery, and both models identified and segmented them, with the Yolov8 model presenting higher accuracy. Lastly, the model trained using Yolov8 could segment most of the panicles and even better distinguish panicles in the background.

### Density maps

The model trained using Sorghum-Net architecture presented lower values of MAE, MSE, and MAPE (26, 1,271, and 17%, respectively) compared to all the tested models (56, 5,350, and 36% for MCNN and 39, 2,821, and 28% for TCNN-seed) (Table 2). However, MCNN required less computational space because it used a smaller file size of model weights and a smaller number of parameters (1.0MB and 78K, respectively) compared to the other models (1.5MB and 113K for TCNN-Seed and 6.9MB and 568K for Sorghum-Net). Despite these distinctions, no significant differences were found for the inference time among the 3 models (~16 ms), which does not represent a bottleneck for future applications.

**Table 2.** Density map models’ comparisons. Comparison of the trained models relative to ground truth data to generate density maps from image patches of segmented sorghum panicles taken at field scale using a smartphone camera.

Model architecture	MAE	MSE	MAPE	Weight file size (MB)	Number of parameters
MCNN (Zhang et al. [35])	56	5,350	36%	1.0	78K
TCNN-Seed (Li et al. [37])	39	2,821	28%	1.5	113K
Sorghum-Net (this study)	26	1,271	17%	6.9	568K

**Fig. 6.**
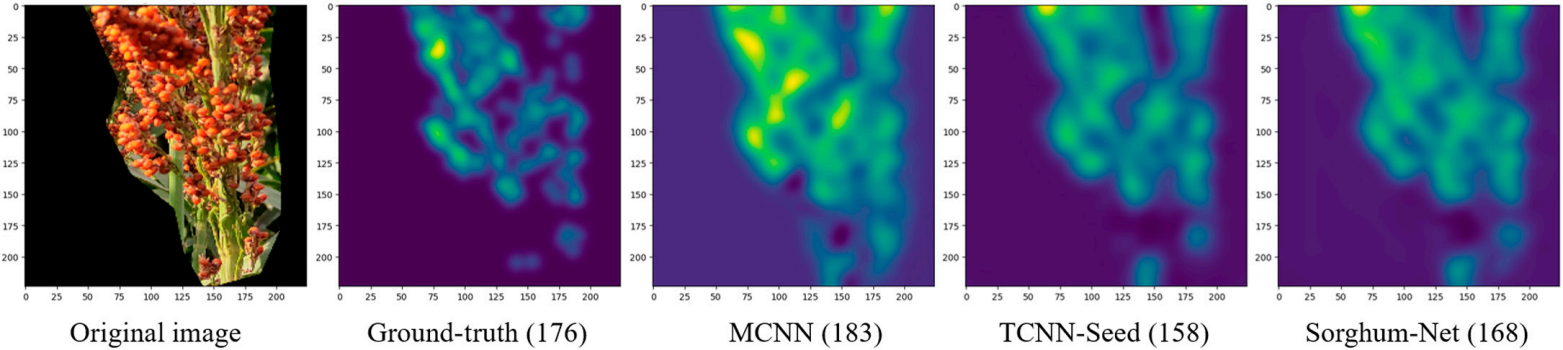
Original image, ground truth, and density map predictions. Original image and density map predictions from the MCNN, TCNN-Seed, and Sorghum-Net models. The number between the parentheses indicates the number of grains present (for the ground truth) and predicted (for the models) in the image.

All models could successfully estimate the overall density of the image, identifying empty spaces and areas of high grain density (Fig. 6). The MCNN model overpredicted areas with high grain density, while TCNN-Seed and Sorghum-Net could reasonably estimate grains with high density. Sorghum-Net showed a more diffuse prediction, indicating more uncertainty in predicting the location of the point. This uncertainty was not a negative aspect as it reduced false negatives (over predictions).

### Estimation of the whole panicle

The observed dataset showed a wide range of grain numbers per panicle, with a minimum of 672 to a maximum of 4,156 grains, with a mean of 2,363 grains for the entire dataset.

**Fig. 7.**
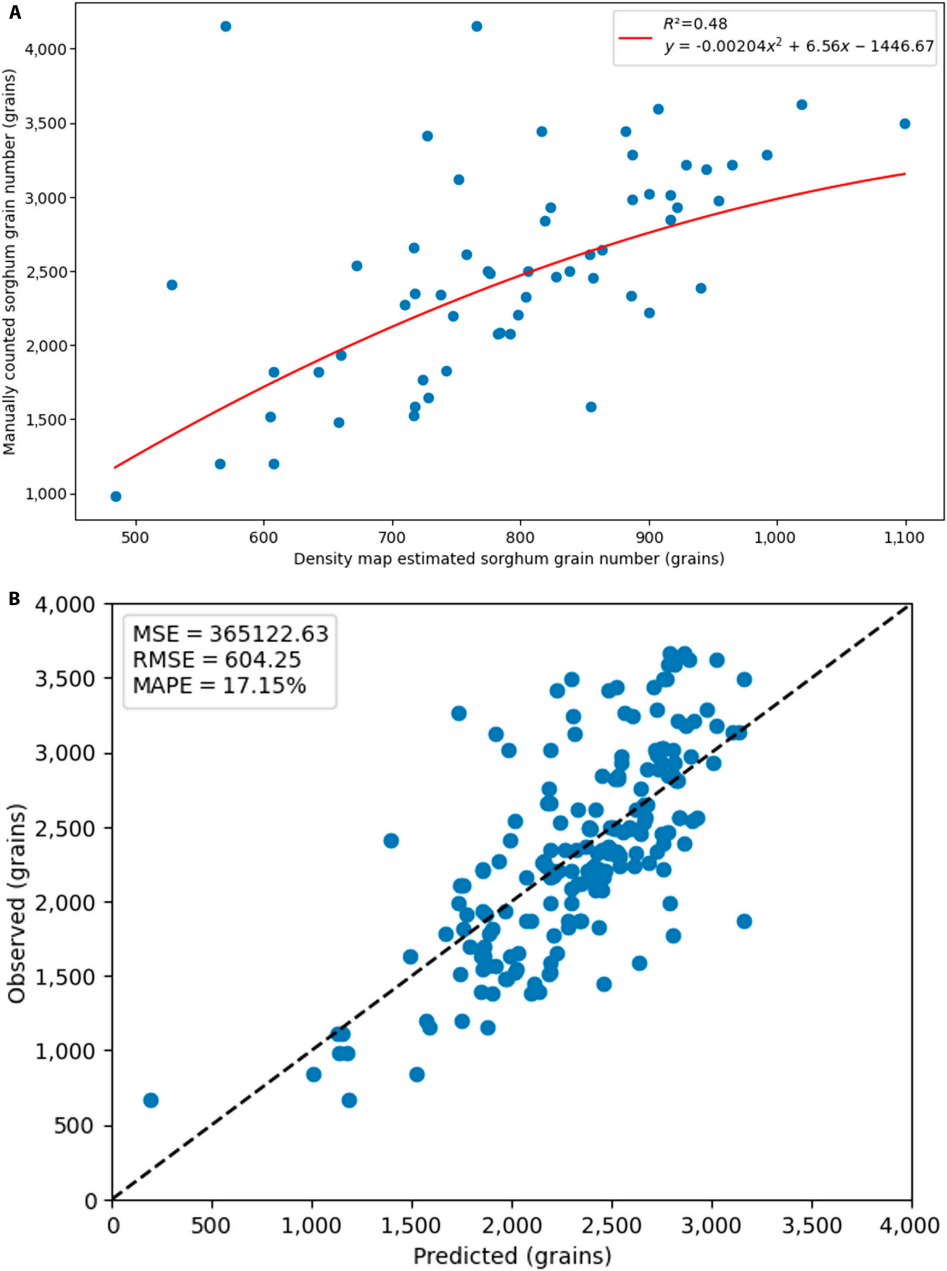
Sorghum grain number estimation via a density map and predicted with independent field data. (A) Proposed polynomial model (fitted model, red line) for predicting total grain number from the output obtained from the density map generated using the Sorghum-Net model (considering only one side of the panicle) and benchmarked against the dataset of the number of grains manually counted of the entire panicle. (B) Observed versus predicted total number of grains of a sorghum panicle.

A polynomial linear model was fitted to predict the total number of grains per panicle using the prediction obtained from the density maps of Sorghum-Net (Fig. 7A). This model showed an *R*^2^ of 0.48, an MSE of 365,122, an RMSE of 604 grains, and a MAPE of 17%. The 1:1 scatter plot showing the observed (ground truth number of grains in the panicles) versus predicted (using the polynomial linear model fitted to the predictions outputted from the density maps) is shown in Fig. 7B.

In addition, predictions made using images from opposite sides of the same panicle differed by 103 grains (RMSE). For this task, the MAE was 74 and the MSE was 10,524. These results showed that the tested sorghum panicles were rather symmetrical, with minor differences in grain number estimation using one image.

## Discussion

### Framework development

This study developed a framework linking CNN and a linear model to accurately estimate the number of grains in a sorghum panicle using images captured by a smartphone camera under field conditions. Methodologically, our study compares the performance of Yolov8 and Detectron2 for detection and segmentation tasks. Similar comparisons have been conducted in other domains [44,45]. Prasetyo et al. [44] compared the performance of these models to segment the head and tail of fish with overlapping issues in the images. Similar to our study, Mask-RCNN presented lower precision and recall. The same comparison was made by Ghafari et al. [45] to detect cells from microfluidic images, where Yolov3 outperformed Mask-RCNN. In these studies, members of the Yolo family outperformed Mask-RCNN, Detectron2’s model architecture for segmentation tasks, in terms of precision and recall when segmenting overlapping objects. Likewise in other research outcomes [44–48], this study also points out that the panicles in the background and foreground panicles share visual similarities, which explains the relatively lower metrics presented in our study, and the overall complexity of unfolding this task under field conditions.

Grain counting, the next step in our framework, presents challenges related to the different scales in the images [35]. The panicle images used in this study, similar to the pod images used by Li et al. [37], presented grains of approximately the same size, which explains the lower metrics achieved by the MCNN model, and the high metrics of TCNN-Seed presented in our study. Sorghum-Net, the model proposed in this work, shows an improved performance, possibly due to its higher complexity, which allows a more effective feature extraction. The metrics and the visual predictions obtained from the density maps showed that the model trained using the Sorghum-Net architecture presented more than half of the error compared to one using the MCNN architecture. Furthermore, the model trained using Sorghum-Net had 1.5 times less error relative to the TCNN-Seed architecture. Therefore, among the tested models, the Sorghum-Net architecture is the best tested option for counting the number of grains within a sorghum panicle using segmented images obtained at the plant level. The size of the weight file and the number of parameters would affect the overall time to complete the predictions depending on the hardware used to perform the computations, but no differences in inference time were observed among the 3 models [49,50]. Lastly, the polynomial linear model predictions based on density models achieved superior accuracy in estimating the number of grains in the entire panicle compared to previous attempts [6,8].

### Comparison with existing technologies

At the plant level, noninvasive attempts to count the number of grains within a sorghum panicle were performed by James et al. [15] using a deep learning method for point cloud feature extraction for grain detection and counting of sorghum panicles. The same authors obtained a MAPE of 6.5%; however, the experiment was performed in a laboratory setting using a high-quality digital camera and a tent to collect images. Using a smartphone camera at the plant level, Santiago et al. [8] obtained a root mean square percentage error of 53%, higher than the one obtained in this study. Using a similar approach, but for maize kernels, Khaki et al. [42] developed DeepCorn, a semisupervised deep learning method, and obtained a mean percent error of 30% and a number of parameters of 26.6 M.

Sorghum high-through phenotyping techniques are also being addressed using UAVs to obtain images. Li et al. [36] compared different deep learning methods to count sorghum panicles; EfficientDet, SSD, and YOLOv4 were used, with the latter showing the best results for the task, with a precision of 98%. Li et al. [51] used deep learning methods to segment sorghum panicles using images obtained from UAVs, with an accuracy of 94%. Despite the aforementioned studies showed excellent results, they did not consider the number of grains within the panicles, which is important information for breeders and yield estimation. UAV and aerial imagery represent the second level of phenotyping, being able to count plants and panicles, not the number of grains.

The use of satellites to predict sorghum yield has also been used. Yang et al. [52] employed satellite imagery to estimate crop yield and obtained an *R*^2^ of 0.8 in the estimated versus measured yield model. Despite the high *R*^2^ obtained, the imagery used had a spatial resolution of 10 m, which did not allow for high-throughput phenotyping at the plant level. Satellite imagery provides a third level of phenotyping, yield estimation at the field or regional level.

Integration of the 3 levels can increase genetic gain by analyzing different levels of phenotyping and crop aspects [53]. In the future, satellite or aerial imagery can be used to guide scouting of sections of interest in the field; during scouting, the plant-level handheld or proximal devices will be used at the plant level [54]. The models presented in this paper provide the cornerstone of these equipment back-ends, counting the number of grains in sorghum panicles, and thus supporting high-throughput plant-level phenotyping.

### Limitations and future applications

The overlap between the background and foreground sorghum panicles presented in the field images led to difficulties in manual labeling and increased false-positive values. Nevertheless, it is important to recognize that this limitation is an inherent setting of the proposed framework, as observed in a similar field study [51].

The continuous improvement of these models is essential to increase their precision and reduce the error metrics [55]. The continuous improvement of these models is essential to increase their precision and reduce the error metrics. Therefore, a future step is to expand the dataset to include images from different lighting conditions, backgrounds, and growth stages. To reduce the effort required to annotate the data, weak supervision training can be used, as proposed by Rühling Cachay et al. and Robinson et al. [56,57]. Performance analysis is fundamental when using CNN models [58]. The size of the weight file and the number of parameters would affect the overall time to complete the predictions depending on the hardware used to perform the computations, but no differences in inference time were observed among the 3 models [59,60].

The grain number estimation step within a sorghum panicle is a critical component in developing effective yield prediction models [5], due to the importance of grain number in yield formation [7]. The implementation of this approach for smartphone devices will assist farmers and breeders in estimating sorghum yield prior to harvest, a crucial advancement in the development of future digital decision support tools. This application promises to provide timely and reliable sorghum yield information to aid macroregulation of prices [61,62] and to support with high-throughput phenotyping for breeders, agronomists, and farmers under field conditions [63]. Lastly, this framework can be adapted to other crops, such as millet and wheat, to provide a comprehensive approach to estimate yields prior to harvest.

In summary, this study addressed a critical bottleneck in advancing field trait characterization and yield estimation for sorghum crops by integrating computer vision and artificial intelligence with traditional field phenotyping. The proposed framework lays the foundation for the development of a robust plant-level application for yield estimation to benefit farmers and breeders. Further refinement and validation are crucial for broader applicability, and the integration into a plant-level application will be a significant step toward using advanced technologies for efficient sorghum yield estimation.

## Data Availability

The data used in this study are available upon reasonable request. The code used to train, test, and analyze the data is available at https://github.com/GustavoSantiago113/Sorghum_Grain_Counter.
